# Population size and human-grivet monkeys (*Chlorocebus aethiops*) conflict in Zegie peninsula, Bahir Dar, Ethiopia

**DOI:** 10.1186/s40850-021-00066-w

**Published:** 2021-03-06

**Authors:** Yibelu Yitayih, Dessalegn Ejigu, Misganaw Mola

**Affiliations:** 1 Mizan Tepi University, College of Natural and Computational Science, Department of Biology, P.O. Box 121, Tepi, Ethiopia; 2 Bahir Dar University, College of Science, Department of Biology, P.O.Box 79, Bahir Dar, Ethiopia

**Keywords:** Grivet monkeys, Human-wildlife conflict, Population, Zegie peninsula

## Abstract

**Background:**

Human-monkey conflict exists in different forms all over the world and is experienced more in developing countries. The conflict between human and grivet monkey (*Chlorocebus aethiops*) ranks among the main threats to biodiversity conservation and has become frequent and severe in different parts of Africa. A study on population size and human-grivet monkey conflict in Zegie Peninsula was carried out from August 2019 to March 2020; the study comprised both the wet and dry seasons. The line transect method was used to collect data on the population size of grivet monkeys. Questionnaires and focus group discussions were used to study the human-grivet monkey conflict and its conservation status.

**Results:**

The estimated population of grivets in the study area was 5046. Population structure of grivets indicated that there were 637 adult males, 1246 adult females, 1839 juveniles, and 1324 infants. The number of grivets in different habitat was; 1925 grivets in agricultural area, 1568 in lakeshore, 988 in forest and 565 grivets in shrub. Grivet population estimate between the wet and dry seasons did not show significant differences (χ^2^ = 0.941, df = 1, *p* > 0.05). But there was a significant difference in the population estimate of grivets among the different habitats (χ^2^ = 239.135, df = 3, *p* < 0.05 and among their age/sex (χ^2^ = 504.102, df = 3, *p* < 0.05.

Based on the questionnaire result the most problematic crop pests in the area were grivet monkeys (96.4%), bush pigs (52.3%), porcupine (46.3%) and squirrels cover (33.2%). The result of discussions held with focus groups and questionnaires in the study area showed that grivet monkeys damage crops (98.4%), chicken depredation (49.5%), steal and snatch human food (32%), disturbing communities (11.3%) in the area. Most (62%) of the respondents noted that guava is the most vulnerable crop followed by papaya, mango, avocado to be damaged by grivet monkeys.

**Conclusion:**

The total number of grivet monkey in the study area is 5046. The number of grivet monkeys varies based on their sex/age and their habitat type. There is no significant difference in their number according to season since the movement of grivets is restricted to the area (there is no migration in the area). The increasing population number leads the occurrence of human grivet monkey conflict in the area and this inversely affects grivet monkeys. There was a human-grivet monkey conflict in the Peninsula and grivets damaged crops, not only crops but also predating poultry, stealing eggs, and human food. Consequently, grivets were killed in response to the damage they caused to crops. Grivet’s habitat in the Peninsula is highly disturbed because the local people cut trees for sale to support their livelihood. Awareness creation of the residents about wildlife and their habitat is necessary, and people should understand the impact of deforestation, illegal hunting, and the role of grivets in the ecosystem at large. Hence there is a need to protect the forest in order to ensure sustainable conservation of biodiversity in general and grivet monkeys in particular.

## Background

Ethiopia has long been recognized for its richness in wildlife resources [1] with a diverse mammalian fauna of 325 species within 52 families [2], of which 57 species are endemic to the country [1]. There are 216 species and sub-species of primates in Africa [3]. Primates occupy a wide range of habitats. In addition, they occupy a wide diversity of ecological niches [4].

Different species and subspecies of primates occur in Ethiopia. These are Bushbaby or Senegal lesser galago (*Galago senegalensis*) and Somali lesser galago (*Galago gallarum*) [5], Hamadryas baboon (*Papio hamadryas*), olive baboon (*Papio hamadryas anubis*), black and white colobus monkey (*Colobus guereza*), gelada baboon (*Theropithecus gelada*), Grivet monkey (*Chlorocebus aethiops*), Black-faced vervet (*Cercopithecus. pygerythrus*), Bale monkey (*Chlorocebus aethiops djamdjamensis*), De Brazza’s monkey (*Cercopithecus neglectus*), Patas monkey (*Erythrocebus patas*), Sykes’ Monkey (*Cercopithecus albogularis*) [6], two subspecies of blue monkey (*Cercopithecus mitis stuhlmanni*) [7] and blue monkeys *Cercopithecus mitis boutourlinii*) [6].

Grivet monkeys are an Old World monkey with long white tufts of hair along the sides of the face [8]. They inhabit a wide range of habitat types including savannah, woodland, forest, grassland, riverine and gallery forests [6]. The grivets need to live around a source of water, especially during the dry season [9]. They are distributed along with the southeastern Sudan, Ethiopia, and Eritrea [6].

Human-Wildlife Conflict (HWC) is commonly described as a conflict that occurs between people and wildlife [10], and actions by humans or wildlife that have an adverse effect on the other [11]. According to the International Union for the Conservation of Nature (IUCN) World Parks Congress, human-wildlife conflict occurs when wildlife requirements encroach on those of human populations, with costs both to residents and wild animals [12]. It tends to manifest itself in scenarios where human strategies affect the free movement of wild animals and vice versa [13].

Human-monkey conflict exists in different forms all over the world and is experienced more in developing countries. The conflict between human and grivet monkey (*Chlorocebus aethiops*) ranks among the main threats to biodiversity conservation and has become frequent and severe in different parts of Africa HWC is intense in developing countries particularly in Africa including Ethiopia, mainly in and around protected areas where humans and wildlife live in proximity [14]. Insects, small mammals, monkeys, baboons, warthogs, elephants, and different antelopes cause major crop damage when these animals venture out of the protected areas looking for food [15].

In many regions, human-wildlife conflicts have intensified over recent decades as a result of human population growth and the related expansion of agricultural and industrial activities. The increasing human population in Ethiopia has resulted in overexploitation of natural resources, which in turn led to a variety of human-wildlife conflict [14]. Consequently, it leads to greater contact and conflict with humans as wild animals seek to fulfill their nutritional, ecological, and behavioral needs [16]. The damage to human interests caused by contact with pests can include loss of life or injury, threats to economic security and reduced food security and livelihood opportunities. A long term solution to human-monkey conflict can only be realized when an in-depth scientific research regarding human-monkey conflict is conducted since it affects the livelihood of fringed communities [17]. The major conflict in Zegie Peninsula is between humans and grivet monkeys [14]. This study is essential for peoples of Zegie and also for grivet monkeys in the study area. This study is mainly aimed to provide information about grivet monkey population structure and human-grivet monkey conflict in Zegie Peninsula, Bahir Dar, Ethiopia.

## Results

### Population estimate

**Fig. 1:**
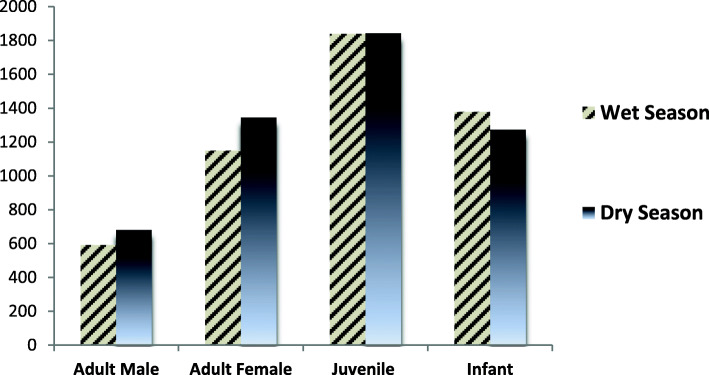
Mean number of grivet monkeys, estimated during the wet and dry seasons

The estimated mean number of grivet monkeys in the whole study area (1150 ha) was 5046. The estimated numbers of grivets during the wet and dry seasons were 4955 and 5138 respectively. The estimated numbers of grivet monkeys in the whole study area were 637 adult males, 1246 adult females, 1839 juveniles, and 1324 infants. The estimated number of grivet monkeys in different sex and age categories during the wet season and the dry season is presented in Fig. 1. There was significant difference in number of individuals among the age groups (x^2^ = 504.102, df = 3, *p* < 0.05). There was no significant difference in the number of grivet monkeys between the wet and dry seasons (x^2^ = 0.941, df = 1, *p* > 0.05).

**Fig. 2:**
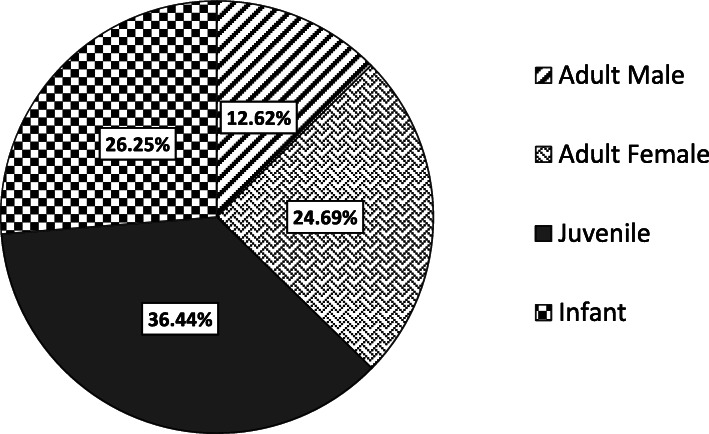
The percentage of grivets in different age and sex categories

The percentage of different age and sex categories from the total number of grivet monkeys in the study area is shown below (Fig. 2).

The estimated number of grivets in different habitats varied with habitat type. The numbers of grivet monkeys in the agricultural area were 1925, in lakeshore 1568, while the forest and shrub habitats comprised 988 and 565 respectively. The number of grivets during the two seasons was 1964 individuals inhabited in the agricultural area, 1322 in lakeshore, 1077 in the forest and 591 in shrub during the wet season, while during the dry season there were 1886 grivets in the agricultural area, 1814 in lakeshore, 899 in forest and 539 in shrub habitat.

From the total number of grivet monkeys in the study area the percentage composition of grivet monkeys in the agricultural area, lakeshore, forest, and shrubs was 38.15, 31.07, 19.59 and 11.19% respectively. There was significant difference in the number of grivet monkeys among the different habitats (x^2^ = 239.135, df = 3, *p* < 0.05).

### Human-grivet monkey conflict

In Zegie Peninsula, occupations of the inhabitants include farming (90.6%), trade (5.9%) and others (3.5%) including wood selling, fishing, artisanship, guiding, cloth sewing and apiculture. The majority of the respondents (66.1%) possess 0.25–1 ha (ha) of land, some others (25.4%) have 1.25–2 ha, (6.5%) have 2.25–3 ha and only (0.9%) of the respondents possess 5 ha farmlands. Among the respondents, 1.1% of the household do not have farmland. Most respondents (97.1%) have poultry, 94.4% of the respondents have sheep, while some of them (1.8%) do not possess domestic animals. In the peninsula coffee, hop, lemon, mango and bitter orange were cultivated by the majority of the inhabitants.

The most problematic crop pests in the area were grivet monkeys (96.4%), bush pigs (52.3%), porcupine (46.3%) and squirrels cover (33.2%). The questionnaire results showed that duiker, bush pig, bushbuck and grivet monkey are the most illegally hunted wild animals for bushmeat or as a result of crop-raiding and predation of domestic animals. Depredation of sheep and domestic dogs by the leopard, chickens by the wild cat and sometimes by grivet monkeys is frequent in the Peninsula. From the results of the questionnaire with the inhabitants most respondents (96.4%) considered grivet monkey as the most problematic wild animal in the area and 52.3% of the respondents described bush pig as the second followed by a porcupine (46.3%) and squirrel (33.2%).

The result of discussions held with focus groups and questionnaires in the study area showed that there were conflicts between the local communities and grivet monkeys in the Peninsula. Most of the respondents noted that grivet monkeys are the most crop pest among wild animals. They replied that grivet monkeys damage crops (98.4%), chicken depredation (49.5%), steal and snatch human food (32%), disturbing communities (11.3%) in the area. Most (62%) of the respondents noted that guava is the most vulnerable crop followed by papaya, mango, avocado to be damaged by grivet monkeys.

### Threats of grivet monkeys

The heaviest impact on grivet monkeys in the study area was likely to be human activities such as habitat destruction and illegal hunting. Most (98.4%) of the respondent described that grivet monkeys are disadvantageous species because of their impact on the livelihood of the local community. The presence of negative attitudes of the local community on grivet monkeys is the main threat to the conservation of grivets in the area. Most respondents carried out different activities like chasing (72.6%), snare (63%) poisoning (24%) and shooting (8%) to avoid grivets in the area.

Among the respondents, 72.7% replied that there was a decline in forest coverage of the area. Cutting trees for firewood, wood sale and timber production are the main causes for declining forest coverage in the Peninsula. These negative activities of the inhabitants destroy the habitats of grivet monkeys and reduce food availability for the species. Because of the scarcity of natural food, grivet monkeys share resources with the local communities. Consequently, there is increasing human-grivet monkey conflict in the study area, and grivets are faced with possibly being killed by the local communities.

## Discussion

In the present study an average 5046 grivet monkeys were recorded in the study area. The number of grivet monkeys varies with the habitat types. In the Zegie Peninsula, large numbers of grivet monkeys are found in the agricultural area during the wet season compared with the dry season, results of a study by Getachew Gebeyehu and Afework Bekele [14]. This might be due to the presence of more edible fruits and vegetables in the agricultural area during the wet season than the dry season.

The number of grivet monkeys is relatively higher in the lakeshore during the dry season than the wet season. This difference might be due to their demand to get more water during the dry season and thus they aggregate closer to lakeshore habitats. The types of plants in the lakeshore and forest habitats are almost similar. However, as the lakeshore habitat is adjacent to the lake, they can get water easily and more grivets are found in lakeshore habitats than forests during the dry season. Thus, depending on their demand for food and water, grivets shift their habitats between the dry and wet seasons. The same is true in shrub habitat as a large number of grivets visit shrub habitat during the wet season than the dry season due to the availability of edible herbs and invertebrates.

Generally, the number of grivet monkeys is higher in the agricultural areas followed by the lakeshore, forest, and shrub habitats. These differences might be due to the variation in food and water availability and/or resting sites, the study is similar with a research conducted by [18]. On average there were about four grivet monkey per hectare in the Zegie Peninsula.

The age structure of grivets showed that adults are more in number, followed by juveniles, while infants are the least. The size of a population and its age and sex composition may indicate its viability [19]. Female biased sex ratio and a fairly high proportion of juveniles indicate a healthy population [20].

In Zelie Peninsula the main occupation of the people was working on a coffee plantation. The respondents acknowledged that until recently there was no farming practice because the monasteries in the Peninsula had forbidden the use of any type of draft animal for farming. Nevertheless, currently, people have started farming and clearing the forest for agricultural purposes and this may affect the natural habitats of some of the wild animals [21].

Zegie Peninsula is the most convenient areas for grivet monkeys as the habitat is characterized by its dense forest with perennial trees and partly enclosed by Lake Tana. The majority of the Old World monkeys of the genus *Cercopithecus* occupy varying forest habitats, from primary, secondary and rain forest to bamboo forest, flooded and swamp forest [22].

The total number of grivet monkeys in the study area is increasing as it is compared to the study conducted before ten years by Getachew Gebeyehu and Afework Bekele [14]. As a result, human-grivet monkeys conflict is a spearhead problem in the area. The majority of the respondents (94.6%) describe that grivets are categorized as the most problematic wild animal in the peninsula. In this area, most of the residents are dependent on cultivating fruits and vegetables. Similarly, the feeding habits of grivet monkeys are mostly dependent on fruits and vegetables [21].inhabitants cannot farm inedible plants and other crops like cereals because of the monastery don’t allow plowing by anima in the area. Consequently, the human-grivet monkey conflict is an unending problem in the area.

In the absence of viable alternative economic activities, many residents of Zegie Peninsula have resorted to cut trees for sale and firewood, the study similar to the idea conducted [20]. According to CARE Ethiopia [23] 90% of the firewood entering Bahir Dar city is from Zegie Peninsula. It is common to get wood collected from the peninsula placed in several places of the lakeshore that is ready to be transported to Bahir Dar by boats. Boats have been carrying wood for sale to Bahir city every day. Similar activities had also been reported by Getachew Gebeyehu and Afework Bekele, [14]. Thus, it is not difficult to understand that grivet monkeys are also faced with a problem as a result of habitat destruction and food scarcity in the study area [24]. Human-grivet monkey conflict in Zegie Peninsula affects the free movement of local communities and grivet monkeys in a similar manner.

Most respondents replied that there is an increasing tendency of crop damage by grivet monkeys from time to time, and this coincides with the report of Getachew Gebeyehu and Afework Bekele [14]. The effect of grivet monkeys is not limited by crop damage but they also steal and snatch human food and eggs from kids and old men, and hunt chicken which is similar to the findings of Dessalegn Ejigu and Afework Bekele [24, 25]. However, such modified feeding behavior of grivets in Zegie Peninsula is not reported by Getachew Gebeyehu and Afework Bekele [14]. The development of adaptive feeding behavior of grivets in Zegie Peninsula might be an indication for limited food available for the species in the area as adopted from Wrangham and Waterman [26].

Most (66.1%) of the respondents possessed 0.25 to 1 ha farmland and they produce a limited number of crops mainly coffee, hop and fruits such as lemon, mango, and others. Most of the crops cultivated by the local communities in the peninsula are vulnerable to be damaged by grivet monkeys. As a result, people in Zegie Peninsula described grivet monkeys as disadvantageous because of the nature of crops cultivated and property damage [14].

To maintain their economy people cut trees for firewood, charcoal and for sale. The residents also produce hop plants (which is inedible by grivet monkeys). Most of the inhabitants produce hop next to coffee. Coffee is the most dominant crop in Zegie Peninsula, and it is a widely cultivated crop and found in the forest covered by canopies of large perennial trees. Grivet monkeys feed on coffee fruit rarely when the fruit is ripen. This study is supported by Tilahun Teclehaimanot and Mirutse Giday [21].

Local people in Zegie Peninsula have used some pest control methods in order to minimize the impact of crop pests on crops. Most crop pest controlling methods are chasing, killing and trapping by snare, the study is similar with the research conducted [27]. During focus group discussion, the residents described that grivet monkey, bush pig, porcupine, leopard and hyena are killed by illegal hunters in the Peninsula, and the same practice has also been reported by Petersen [15] and Mudingu [28] in different habitats. During the study period, the corpse of a wild cat that was killed by the resident was observed. Wild cats are well known for depredating poultry in the area.

## Conclusion

In the present study, there are about 5000 grivet monkeys residing in the Peninsula, and their number varied based on their sex and age. There are a large number of adults followed by juveniles and infants. The population size of grivet monkeys varied among the different habitat types. The human-grivet monkey conflict is an urgent issue in Zegie Peninsula. It is highly increasing from time to time. The overlapping of the home range of grivet monkeys with human settlement makes the conflict sever. The similarity between types of crop cultivated in the peninsula and favorite food of grivet monkeys could also be considered as the main causes for increasing human-grivet monkey conflict in the study area. Anthropogenic activities including trapping by snare and killing of wild animals to control their crops were the main threats for wild animals in general and grivets in particular. To ensure their livelihood local people have resorted to cut trees for sale and firewood. This activity causes habitat destruction and food scarcity for grivet monkeys and other wild animals in the peninsula.

The results of the present study have several conservation and management implications for wild animals and their habitats. Zegie Peninsula is severely threatened due to various anthropogenic activities. The government should take action to prohibit deforestation. Cutting trees for sale and for firewood has a significant impact in the area in accelerating habitat destruction and human-grivet monkey conflict. Improving awareness among the local community might be the possible solution to alleviate the current problems in the study area.

Isolating wild animal habitat from human settlement might be the most effective solution for both human and wild animals. To settle the human-grivet monkey conflict, people may avoid their dependence on fruit crops that attracted more wild animals and cultivate others that are not attractive instead.

Awareness should be created among the inhabitants about the importance of wildlife and the negative consequences of illegal hunting and deforestation. The government should involve community leaders, monks and indigenous peoples to construct conservation strategy of natural resources in the area. The government also takes appropriate action to stop the trapping and killing of grivet monkeys and other wild animals in the area. Therefore, proper law enforcement should be implemented to prohibit the destructive anthropogenic activities on wild animals as well as their natural habitats.

## Methods

### Description of the study area

**Fig. 3:**
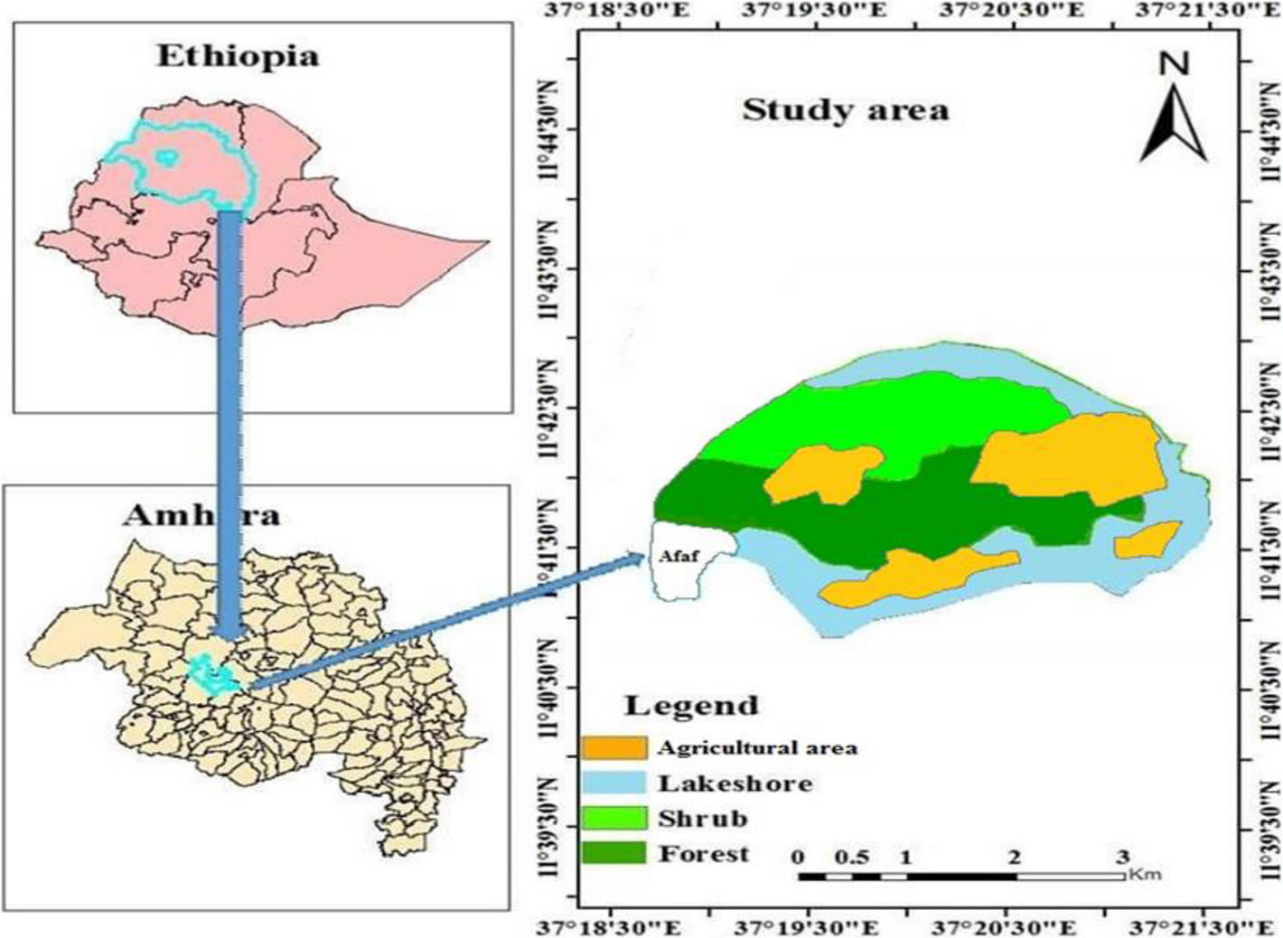
Location map of the study area

The study was conducted in Zegie Peninsula which is the largest peninsula along Lake Tana. Zegie Peninsula is situated at 11° 40′ to 11° 43′ N latitudes and 37 °19′ to 37 °21′ E longitudes, an average altitude of 1800 m asl, and found at 600 km northwest of Addis Ababa. It is partly surrounded by Lake Tana, the largest lake in Ethiopia. Zegie is part of Bahir Dar City Administration and is 31 km far away from Bahir Dar town. It includes a town called Zegie (Afaf) and two rural Kebeles, Ura and Yiganda with an area of 1347 ha. The study focuses on the two rural Kebeles Ura and Yiganda it covers the total area of 1150 ha (ha) (Fig. 3).

Zegie lies in the moist ‘Woina Dega’ agro-climatic zone and it is covered by dense forest trees and shrubs. The mean minimum and maximum temperatures for 10 years (2008–2018) of meteorological data indicate 12.1 °C and 28.43 °C, respectively, with the average annual rainfall of 1726.07 mm (ENMASNWS).

Wild animals such as grivet monkey, porcupine, Nile monitor, jackal, bush pig, hyena, leopard, duiker, squirrel, hare and bushbuck are common in the peninsula [14]. Zegie Peninsula possesses one of the very few remaining virgin tropical forests in Ethiopia [23]. The most dominant trees include *Mimuscops kummel, Ficus sur, Strychanoss pinosa, Ficus vasta, Cordia africana, Capparis tomentosa, Prunus africana, Croton marcostachyu, Albiza gummifera, Jacaranda mimosifolia, Millettia ferruginae, Podocarpus gracilis, Ficus thonningii, Acacia busseai* and *Grewia ferruginea*.

Livestock rearing, agricultural activity, sheep rearing, poultry production, Charcoal and fuelwood collection are common practices by most of the inhabitants [14]. The economy of Zegie Peninsula revolves around coffee production [23]. However, at present, most families cultivate hop (*Rhaminus pyrinoids*), lemon (*Citrus limon*), citron (*Citrus medica*), orange, bitter orange and sour orange (*Citrus aurantium*), papaya (*Caraca papaya*) and guava (*Psidium guajava*). Few families also grow maize (*Zea mays*), mango (*Mangifera indica*) and avocado (*Persea americana*) [14]. Most of the fruit crops in this area were introduced by CARE Ethiopia in the 1990s [14].

### Sampling design

**Plate 1:**
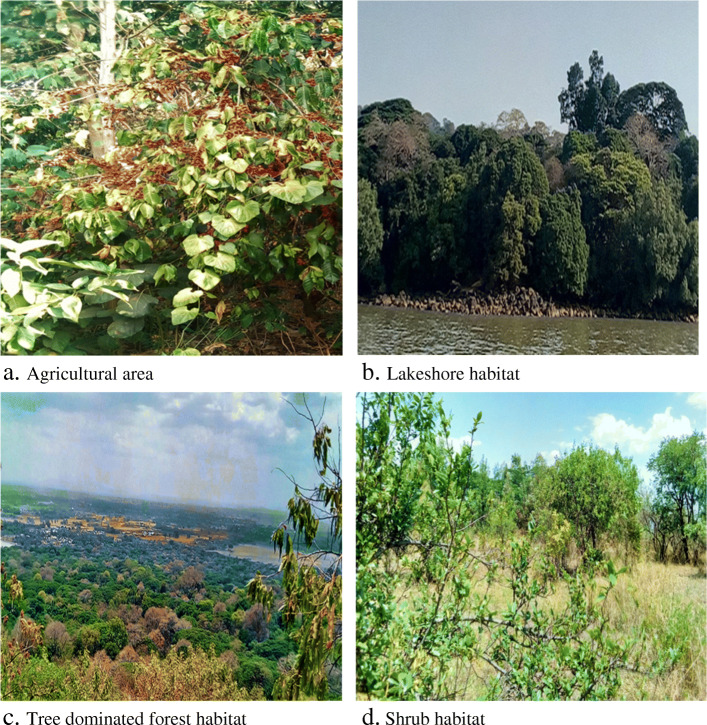
Different types of habitat in the study area

Based on reconnaissance surveys, sample sites were identified and habitat types were classified based on the dominant vegetation type. The site was classified into agricultural area (363.8 ha), lakeshore (328.4 ha), forest (260.7 ha), and shrubs (197.1 ha) (Plate 1). The sample site had an area of 200 m X 500 m (10 ha) each. Out of the total extent of the study area, 40 ha (3.5%) was covered in the four sample sites. Instantaneous scan sampling methods was used to collect data on grivet monkeys [29].

### Data collection

The present research was conducted from August 2019 to March 2020 covering both the wet seasons (August, September, October), and the dry seasons (January, February and March). A preliminary survey was conducted in August 2019 to gather information about the habitats, weather conditions, accessibility, fauna, flora, and topography of the study area.

Population census along line transect designed for forest primates was employed [30, 31]. Transects were placed by a stratified random sampling approach in which transect placement was proportional to the area of different habitats [31, 32]. Five transects with a total length of 0.5 km each were located randomly. Permanent transect lines were delineated by poles and/or natural markers. Each transect was counted four times per month with a total of 24 times, during both the wet (August–October 2019), and dry (January –March 2020) seasons. During transect walks, when grivet monkeys and other animals were encountered, the observer recorded the dominant habitat type where the grivets are spotted, along with the GPS location, time, number of grivet monkeys, sex and age of grivets as adapted from Chiarello, [33] and Fashing and Cords, [34].

Population estimation from line transect sampling is dependent upon the following assumptions [35] including animals on transect are never missed, animals do not move before being seen or flushed and none is counted twice. During the study, transects were covered systematically with a slow speed to maximize the probability of seeing all animals on the transect.

During transect walking all grivet monkeys on the line or immediately adjacent to transect were recorded. Grivet monkeys were categorized by their sex into adult male, adult female and based on their age into adults, juveniles, and infants. To identify their sex and age, different physical characteristics were used. The blue scrotum of adult males was visible from a distance. A pair of nipples in adult females on the chest region and infants sometimes clinging on the belly or on foot helped to identify females [14]. Individuals without infants that had nipples were also classified as adult females. Then, the mean number of grivet monkeys in each sample site was used to estimate the total number for the whole area of each habitat. The sum number of grivet monkey in the four habitats gives the total number of grivet monkeys in the study area. The overall population density of grivet monkey was calculated by dividing the total population number of grivet monkeys by the total study area.

Human-grivet monkey conflict was studied from questionnaire surveys and focus group discussion in localities of Zegie Peninsula from January 2020 to April 2020. A questionnaire was prepared and administered to a total of 339 local residents of 2918 households. The questionnaire involved peoples who have age more than 18 years old. Focus group discussion was carried out with six adult individuals their age is more than 30 years old from the two kebeles (Ura and Yiganda). The questionnaire consisted of both open-ended and fixed-response questions with the following four main categories: personal information, household economy, conflict with grivet monkeys and long term conservation strategy of grivet monkeys. Of the 2918 households, 11.6% (*n* = 339), 197 male and 142 female were selected. All methods were approved by Bahir Dar University Licensing Committee.

### Data analysis

Data were analyzed using SPSS computer program version 23 and Excel software. Descriptive statistics and Chi-square tests were employed to compare the significant difference among variables at 95% confidence interval.

### Ethical statement

The study was approved by institutional review board (IRB) guideline and regulation of college of Sciences at Bahir Dar University, Ethiopia. Permission for the study to be conducted was also obtained from office of Zegie kebele and from Bahir Dar city Administration office. Participants/respondents received explanations of the study and of the purpose of the study in their mother tongue. Informed consent was obtained from study participants before the commencement of each questionnaire and group discussion; and no personal identification was registered. We prepared an informed verbal consent that involved purpose of the research, expected duration of the questionnaire and focus group discussion, and a description that the participants could withdraw from the questionnaire and discussion at any time had no risk and no payment for their recruitment. We read this statement to each study participants before conducting the questionnaire/discussion and requested their permission to be involved in the study.

Their responses had no personal, social or political consequences. We believed that there would not be significant risks to the participants. The IRB approved the proposed verbal consent procedure. The Confidentiality of the data was ensured and access to raw data was allowed only after a joint agreement by the investigators involved in designing, conducting and financing the study.

## Data Availability

The data used and analyzed during the current study is available in the hand of corresponding author for further request if request is available from reviewers without disclosure of the interviewees.

## Abbreviations

GPS: Global Positioning System
HWC: Human Wildlife Conflict
IUCN: International Union for the Conservation of Nature
ENMASNWS: Ethiopian National Metrology Agency Service North West Station
